# A rare case of chronic genian fistula with oral mucosal hair follicle inclusion: case report and review of the literature

**DOI:** 10.3389/froh.2025.1714875

**Published:** 2025-11-05

**Authors:** Edmond D. Ciora, Mariana I. Miron, Andreea Igna, Andreea G. Miron, Ciprian I. Roi, Mircea Riviș

**Affiliations:** 1 Faculty of Dentistry, University Clinic of Oral Rehabilitation and Dental Emergencies, “Victor Babes” University of Medicine and Pharmacy, Timisoara, Romania; 2 Interdisciplinary Research Center for Dental Medical Research, Lasers and Innovative Technologies, “Victor Babes” University of Medicine and Pharmacy, Timisoara, Romania; 3 Faculty of Dental Medicine, Pediatric Dentistry Research Center, University Clinic of Pediatric Dentistry, “Victor Babes” University of Medicine and Pharmacy, Timisoara, Romania; 4 Faculty of Medicine, “Victor Babes” University of Medicine and Pharmacy, Timisoara, Romania; 5 Pathological Anatomy Resident, Timisoara Municipal Emergency Clinical Hospital, Timisoara, Romania; 6 Research Center of Dento-Alveolar Surgery, Anesthesia and Sedation in Dental Medicine, University Clinic of Anesthesiology and Oral Surgery, “Victor Babes” University of Medicine and Pharmacy, Timisoara, Romania

**Keywords:** case report, rare case, oral hair growth, genian fistula, orocutaneous, non-odontogenic

## Abstract

**Background:**

This report describes a rare case of chronic genian fistula associated with inclusion of oral mucosal hair follicle. Ectopic hair growth in the oral cavity is extremely rare and is typically associated with grafting techniques. The presence of a chronic extraoral fistula with intraoral hair follicle inclusion is exceedingly uncommon and poses diagnostic and therapeutic challenges.

**Case presentation:**

A 73-year-old male presented to the Oral and Maxillofacial Surgery Department in Timișoara (Romania) with a chronic cutaneous fistula in the right lower cheek region, unresponsive to antibiotics and without signs of dental or sinus origin. Clinical examination revealed a 0.5 cm cutaneous opening and a filamentous, hair-like structure emerging intraorally from the right maxillary alveolar crest. A submucosal fibrotic tract was palpable, and although the fistula was not clearly visible on CBCT due to its soft tissue nature, imaging confirmed the absence of bony or odontogenic involvement. Given the clear clinical presentation, surgical excision was performed under local anesthesia. Intraoperatively, a keratinized, hair-bearing tract traversing the cheek to the alveolar process was excised *en bloc*. Healing was uneventful, and no recurrence was observed at 14 days.

**Conclusion:**

This is one of the few reported cases of a genian fistula with intraoral hair follicle inclusion in a patient without prior facial surgery. The case highlights the importance of integrating clinical, radiologic, and histopathologic findings in diagnosing and managing rare fistulous tracts.

## 1 Introduction

Oral cutaneous fistulas (OCFs) are abnormal communications between the oral cavity and the skin, most commonly resulting from chronic odontogenic infections or osteomyelitis. These fistulas typically arise from untreated periapical pathology, allowing purulent drainage to follow the path of least resistance and discharge externally through facial or cervical skin. The clinical presentation may be deceptively subtle, often mimicking dermatologic conditions such as epidermoid cysts, furuncles, or even neoplasms (1).

While OCFs themselves are relatively uncommon, the presence of ectopic hair follicles within the oral mucosa is an extremely rare phenomenon. The first documented case was reported by Miles in 1960, who identified a mature hair follicle embedded in the cheek mucosa of an otherwise healthy individual (2). Since then, only a handful of case reports have been published. Baughman and Heidrich (1) described a solitary hair growing from the buccal mucosa, confirmed histologically as a hair follicle within an epithelialized tract (3). In a more recent report, Agha-Hosseini et al. (6) documented intraoral hair in a female patient, suggesting dermoid origin or embryonic inclusion as plausible mechanisms (4).

Most heterotopic hair growths in the oral cavity have been attributed to congenital ectodermal inclusions (e.g., dermoid cysts) or, less commonly, to iatrogenic transposition of hair-bearing skin during reconstructive surgery. A notable example is the case reported by Nair and Beena (7), where intraoral hair growth occurred after flap reconstruction using hair-bearing donor skin (5). Unlike their surgically induced case, the patient in our report had no history of intraoral surgery, indicating a non-iatrogenic, possibly congenital origin of the epithelialized, pilose tract.

This report contributes to the limited literature by describing a chronic genian fistula with intraoral keratinized hair in an elderly, polymorbid patient. It highlights the diagnostic complexity of such lesions and underscores the need for a broad differential diagnosis and a multidisciplinary approach when evaluating chronic facial fistulas without clear odontogenic or sinus origin.

## 2 Detailed case description

### 2.1 Patient history and medical background

**Figure 1:**
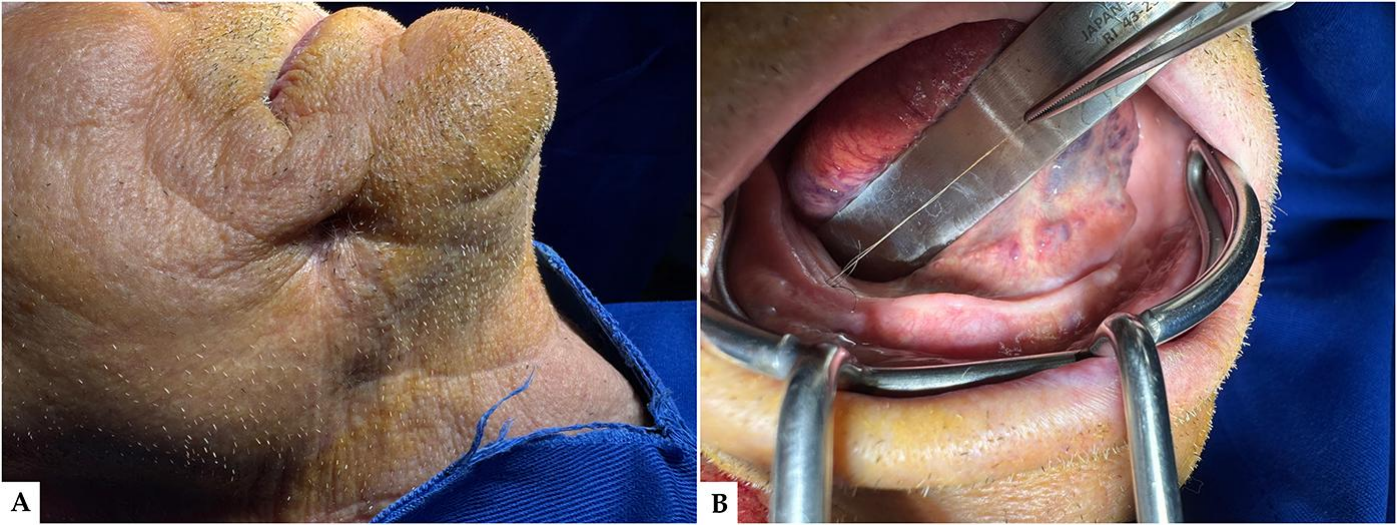
**(A)** Extraoral clinical presentation prior to surgical intervention: a cutaneous opening is clearly visible on the right mandibular region, consistent with a chronic genian fistula; the overlying skin appears adherent and retracted, suggesting long-standing drainage and possible underlying tract formation. **(B)** Preoperative intraoral view: intraoral examination reveals the presence of a protruding hair structure arising from the floor of the mouth, indicative of a transcutaneous fistulous pathway, and the surrounding mucosa presents signs of chronic irritation and fibrosis.

We report the case of a 73-year-old male patient who presented to the Oral and Maxillofacial Surgery Department in Timișoara with a chronic cutaneous fistula located in the right genian (cheek) region (Figure 1). The patient described a several-month history of persistent purulent drainage and intermittent local inflammation in the affected area, unresponsive to repeated empirical antibiotic therapy.

His medical history was notable for a complex polymorbid profile. He had insulin-dependent type II diabetes mellitus with acceptable glycemic control under therapy, ischemic heart disease, and a documented history of squamous cell carcinoma of the lung previously treated with combined radio-chemotherapy and immunotherapy. Additional comorbidities included autoimmune thyroiditis with clinical hypothyroidism, chronic anemia, gallbladder lithiasis, and hiatal hernia. The patient was also under chronic antithrombotic therapy, which required perioperative consideration.

### 2.2 Clinical examination

Extraoral clinical evaluation revealed a small, well-demarcated cutaneous opening measuring approximately 0.5 × 0.5 cm in the right lower genian region right above the lower border of the mandible, with occasional seropurulent discharge. The surrounding skin appeared retracted and mildly fibrotic, suggesting a chronic process (Figure 1A). Intraoral inspection revealed an unusual finding: a fine, hair-like filament emerging from the posterior right mandibular alveolar crest. The buccal mucosa overlying the tract was thickened and fibrotic, and the submucosal pathway of the tract could be palpated with mild tenderness (Figure 1B). There were no signs of dental infection, periodontal involvement, or maxillary sinus disease on clinical examination.

**Figure 2:**
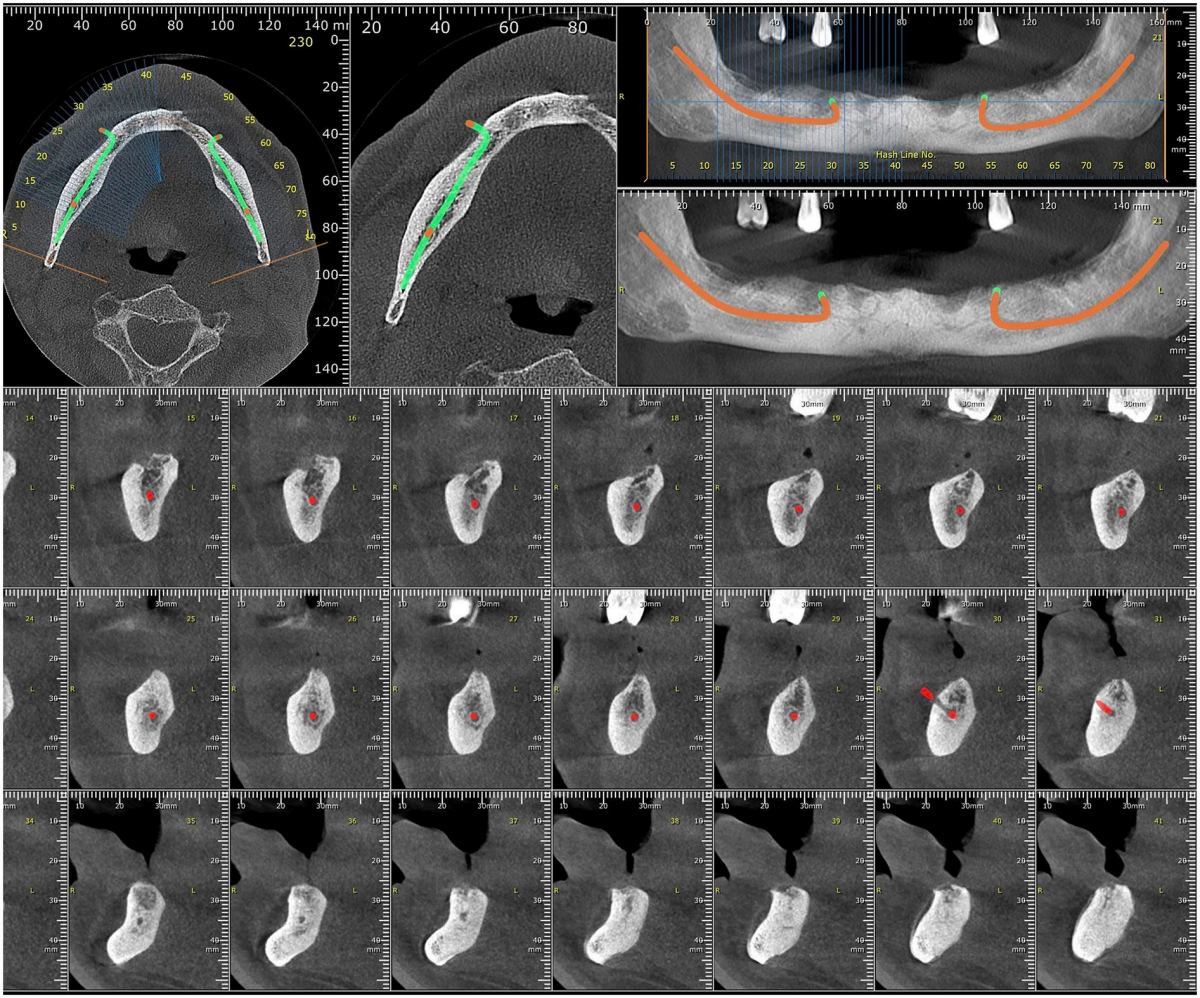
Cone-beam computed tomography (CBCT) axial and cross-sectional views showing absence of bone involvement and superficial course of the fistulous tract (not directly visualized due to soft tissue nature).

Cone-beam computed tomography (CBCT) was performed to evaluate the region of the fistula and adjacent mandibular structures. Although the fistulous tract could not be visualized due to its soft tissue composition, the scan allowed for excluding periapical pathology, bone involvement, or cortical perforation (Figure 2).

### 2.3 Surgical management

**Figure 3:**
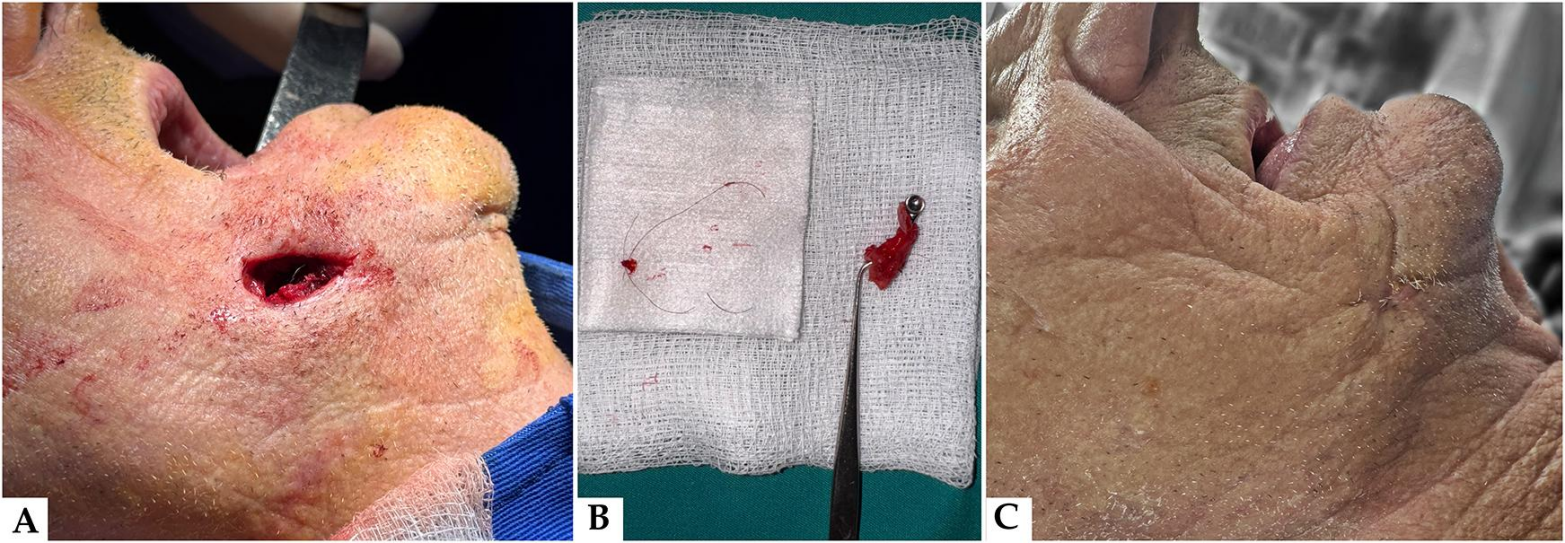
**(A)** Intraoperative view immediately post-excision: the clinical image taken immediately following surgical excision of the fistulous tract reveals the depth and direction of the lesion, confirming communication between the oral cavity and the skin surface. **(B)** Macroscopic view of the excised specimen: fistulous tract with hair follicle. The resected tissue includes the complete epithelialized fistulous tract with an embedded hair follicle, confirming the clinical diagnosis and validating the chosen surgical approach. **(C)** Postoperative outcome: healed extraoral site. Follow-up image illustrating favorable healing of the external skin surface following complete excision of the fistulous tract. No signs of residual infection, drainage, or recurrence were observed at the 14-day postoperative control.

Surgical intervention was performed under local anesthesia using articaine with epinephrine (Septanest 1:200,000). Following aseptic preparation and field isolation, a fusiform excision was made to encompass the cutaneous opening. Careful dissection was carried out along the tract, which was found to be fibrotic, epithelialized, and extending from the skin surface through the subcutaneous tissue to the posterior maxilla (Figure 3A). A complete *en bloc* excision of the tract was achieved. Intraoperatively, the presence of a keratinized, hair-bearing channel was confirmed, traversing the mucosa and soft tissue and terminating in the alveolar process. The specimen, which included the embedded pilose structure, was preserved for histopathological analysis (Figure 3B). The surgical field was irrigated thoroughly and closed in layers using resorbable sutures intraorally and monofilament non-resorbable sutures for the extraoral skin.

**Figure 4:**
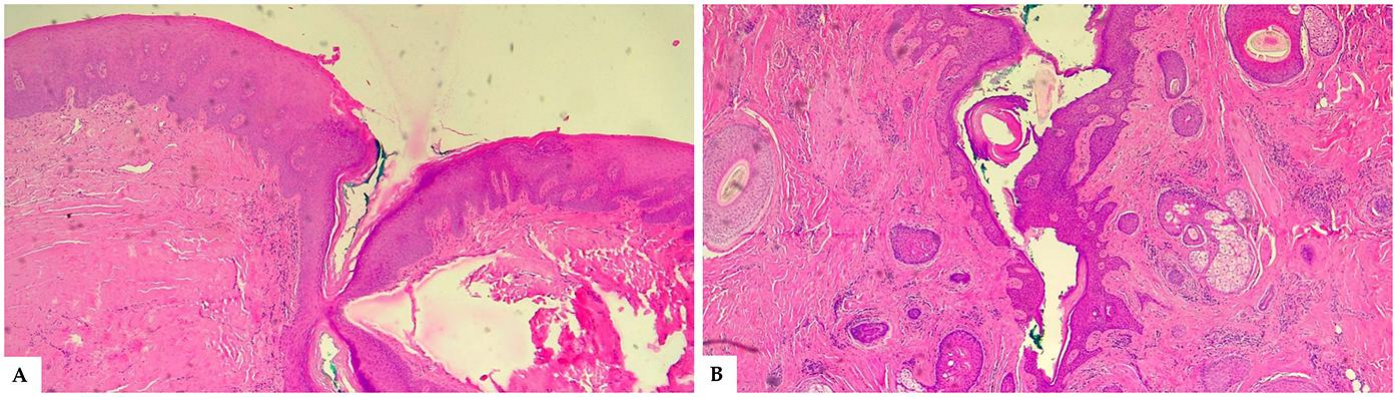
**(A)** Histological section (H&E stain, 4× magnification) showing the superficial opening of a cystically dilated follicular structure communicating with the epidermal surface. The follicular lining is composed of stratified squamous epithelium, and the surrounding dermis exhibits mild inflammatory changes. **(B)** Histological section (H&E stain, 4× magnification) showing the distal portion of a cystically dilated follicular structure filled with concentric keratin debris. The cyst wall demonstrates digitiform epithelial projections extending into the surrounding dermis. Numerous skin adnexal structures are visible: a well-formed hair follicle with its sheath (top right), a sebaceous gland (bottom right), and an additional hair follicle (left side). A mild lymphoplasmacytic inflammatory infiltrate is present throughout the periadnexal connective tissue, visible as scattered purple-staining cells.

The excised tissue was submitted for histopathological examination. The specimen revealed a dermal lesion characterized by cystic dilation of follicular structures in communication with the epidermal surface (Figure 4A). The cyst wall was lined by stratified squamous epithelium exhibiting surface keratinization and focal acanthosis, along with digitiform projections extending into the surrounding dermis. The infundibular lumen contained keratin filaments and hair shaft fragments. A mild lymphoplasmacytic inflammatory infiltrate was observed in the perifollicular connective tissue, arranged in a diffuse pattern (Figure 4B).

The differential diagnosis included odontogenic fistula, dermoid sinus, and post-surgical epithelial inclusion. Odontogenic causes were excluded due to the absence of dental pathology, periapical lesions, or communication with the alveolar ridge. Dermoid sinus was unlikely given the superficial tract, lack of deep extension or midline involvement, and absence of cystic components. Post-surgical inclusions were ruled out based on the patient's history, which revealed no prior surgical intervention in the affected area.

### 2.4 Postoperative outcome

The initial postoperative course was uneventful, with complete closure of the fistulous tract observed at the 14-day follow-up (Figure 3C). The patient continues to be monitored within our hospital system due to multiple comorbidities and has presented for regular medical visits. At the 6-month postoperative follow-up, clinical examination confirmed complete healing, with no evidence of recurrence, inflammation, or scarring at the surgical site. Long-term follow-up remains ongoing.

A concise summary of the clinical chronology, diagnostic investigations, and treatment steps is presented in Table 1.

**Table 1:** Timeline of clinical presentation, diagnosis, and management.

Time point	Event/intervention	Findings/notes
Several months before presentation	Onset of recurrent swelling and purulent discharge in in the right lower cheek region	Patient self-managed with empiric antibiotics prescribed by general practitioner, without improvement
Initial presentation (Day 0)	Evaluation at Oral and Maxillofacial Surgery Department	Chronic cutaneous opening (0.5 × 0.5 cm) on right cheek; intraoral filamentous structure resembling hair emerging from alveolar crest; no dental or sinus pathology identified
Diagnostic stage	Cone-beam computed tomography (CBCT)	No bone involvement or odontogenic origin; superficial soft tissue tract suspected
Surgical intervention (Day 7)	Local excision of epithelialized tract with hair follicle inclusion	Complete *en bloc* resection achieved; specimen sent for histopathological analysis
Histopathological report (Day 14)	Microscopy revealed keratinized squamous epithelium with sebaceous glands and hair follicles	Findings consistent with ectodermal inclusion/dermoid sinus–like lesion
Postoperative follow-up (2 weeks)	Clinical evaluation	Sutures removed; site healed without signs of recurrence; uneventful recovery
Follow-up (6 months)	Long-term outcome	No recurrence, inflammation, or residual scarring; patient asymptomatic

## 3 Literature review and discussion

Ectopic hair growth in the oral cavity is an extremely rare clinical entity, with only a few documented cases in the literature. Reports by Miles (2), Baughman and Heidrich (1, 3), and Agha-Hosseini et al. (4, 6) describe isolated intraoral hair without associated fistulas. Rochefort et al. (6) also described a rare case of a heterotopic hair growing from the dorsum of the tongue in a healthy adult, recurring repeatedly until surgical excision of the follicle was performed. Most reported cases of oral cutaneous fistulas (OCFs) typically arose from chronic odontogenic infections such as untreated periapical lesions, osteomyelitis, or trauma involving the jaws (2). They typically represent a pathological communication between the oral cavity and the skin, most commonly affecting the mandibular region. The cutaneous manifestation often appears distant from the odontogenic origin, leading to frequent misdiagnosis.

Table 2 summarizes the relevant published case reports and literature on intraoral hair growth and orocutaneous fistulas, with focus on symptomatology and clinical outcomes. A focused literature search was carried out in July 2025 using the following electronic databases: MEDLINE via PubMed, Web of Science, EMBASE, Scopus, ScienceDirect, and Google Scholar. The keyword combination used was as follows: “(oral OR intraoral OR buccal) AND (hair OR hair follicle OR pilose) AND (fistula OR sinus OR orocutaneous) AND (case report OR literature review)”. The review was restricted to articles published in English, with full-text access, from 1960 to 2025. We followed an adapted PICOS framework for inclusion: (P) patients with intraoral or orofacial lesions; (I) clinical ob-servation or surgical excision; (C) differential diagnosis with odontogenic infections; (O) confirmation of ectopic hair or epithelialized tract; and (S) case reports or literature reviews. Exclusion criteria were as follows: (a) reports involving hair transplanted via reconstructive surgery unless clearly documented; (b) studies without histological confirmation of hair follicle; and (c) non-English papers without reliable translation. A total of nine publications met our criteria and are summarized in Table 2. These cases vary in anatomical site, underlying cause (congenital, iatrogenic, or odontogenic), and outcome, with our case representing a rare, spontaneous, non-surgical occurrence of intraoral hair within a chronic genian fistula tract.

**Table 2:** Studies on the clinical symptomatology and outcomes associated with genian fistulas and ectopic hair follicles (ordered by relevance to the present case report).

Authors	Year	Paper Ref.	*n*, *N*, Age, Gender	Study design	Symptomatology	Outcome
Baughman et al.	1980	(1)	*n* = 1 45 y, M	Case Report	Hair emerging from attached gingiva near mandibular cuspid	Excision; histology confirmed hair with sebaceous complex
Miles et al.	1960	(2)	*n* = 1 57 y, M	Case Report	Hair follicle in cheek mucosa found at autopsy	Confirmed by histology; noted as unique anomaly
Rochefort et al.	2016	(3)	*n* = 1 30 y, M	Case Report	Hair on dorsal tongue, recurring after removal	Surgical excision; histology confirmed follicle
Sammut et al.	2013	(4)	*N* = 5 21–60 y, M,F	Case Series	Cutaneous sinus tracts due to dental origin	Resolved post endodontic or surgical dental treatment
Lakshmi et al.	2024	(5)	*n* = 1 67 y, M	Case Report	Intraoral hair and orocutaneous fistula without surgery	Fistulectomy and dental extraction planned
Agha-Hosseini et al.	2007	(6)	*n* = 1 11 y, M	Case Report	Multiple intraoral hairs in a boy	Histology showed hair-like structures; biopsy performed
Nair and Beena	2022	(7)	*n* = 1 49 y, M	Case Report	Hair on buccal mucosa post flap surgery	Planned diode laser epilation
Prabhu and Al Abdulla	2020	(8)	*n* = 1 33 y, M	Case Report	Recurrent hair in floor of mouth (Wharton's duct)	Hair removal and stone extraction; no recurrence
Cansiz et al.	2016	(9)	N/A	Literature Review/Chapter	Various oral fistulas (dentoalveolar, orocutaneous, etc.)	Surgical treatment recommendations

F, female; M, male; n, number of cases; N, total number of subjects; y, years.3; N/A, not applicable.

In our case, the patient presented with a persistent genian fistula associated with a pilose (hair-bearing) tract, an exceptionally uncommon presentation. A similarly rare case was described by Lakshmi et al. (5), where a persistent orocutaneous fistula in the buccal vestibule was associated with intraoral hair growth in the absence of prior surgical grafting, suggesting post-traumatic displacement or developmental heterotopia. As described by Chouk and Litaiem (10), most OCFs tend to follow the path of least resistance and drain through the overlying skin, usually without intraoral symptoms (10). Compared to our case, where the fistulous tract extended from the posterior maxilla and emerged intraorally through the alveolar crest, classical presentations more frequently originate from the mandibular molars (10).

Epidemiologically, Guevara-Gutiérrez et al. observed that OCFs are more frequent in older individuals and slightly more common in females (11). While our patient fits the older age group, the maxillary origin and presence of ectodermal elements such as a hair follicle are unusual and sparsely described in the literature.

The presence of a keratinized, hair-bearing tract raises the differential diagnosis of a dermoid sinus or ectodermal inclusion cyst. The histopathologic findings in our patient, characterized by a dilated epithelial tract lined with keratinized squamous epithelium and containing sebaceous glands and hair follicles, share partial similarities with those reported in dermoid cysts of the oral cavity. Patel et al. (12) described a 17-year-old female with a sublingual dermoid cyst exhibiting identical adnexal structures and keratin debris, confirming its ectodermal origin and benign, developmental nature (12). Although our case demonstrates comparable epithelial differentiation, the absence of a cystic lumen and the presence of a superficial epithelialized tract extending to the cutaneous surface distinguish it from a true dermoid cyst. Furthermore, the patient's advanced age and complex systemic background, including diabetes, cardiovascular disease, and previous oncologic therapy, suggest a chronic, possibly acquired mechanism rather than a congenital developmental origin. Collectively, these aspects support the interpretation of a dermoid sinus–like or ectodermal inclusion variant that secondarily fistulized through the genian tissues.

A similar case was reported by Nair and Beena (7), where postoperative hair growth was observed in the oral cavity following reconstructive flap surgery using hair-bearing skin (7). Unlike their surgically induced case, our patient had no prior intraoral surgery, suggesting a congenital or developmental anomaly that had remained epithelialized and had been maintained via chronic low-grade inflammation.

Diagnostic delay and mismanagement are frequently reported. In the series by Lee et al., over 80% of OCFs were initially misdiagnosed as dermatologic lesions such as epidermal cysts or basal cell carcinomas (13). Similarly, Sammut et al. (4) emphasized that nearly half of affected patients undergo prolonged antibiotic therapy or dermatologic procedures before an accurate diagnosis is established, reflecting a persistent clinical challenge (4). Our patient also underwent prolonged empirical antibiotic therapy and was only referred for surgical evaluation after months of unsuccessful treatment. Furthermore, his prior diagnosis of pulmonary squamous cell carcinoma complicated the differential diagnosis, raising concerns about cutaneous metastasis or paraneoplastic phenomena; these scenarios were also discussed by Cansız et al., who described an orocutaneous fistula in a post-radiotherapy patient with oncological history (9).

In this context, the patient's multiple systemic comorbidities, including type II diabetes, cardiovascular disease, hypothyroidism, and a history of cancer therapy, may have contributed to the chronicity and atypical presentation. Diabetes mellitus is known to impair wound healing and modulate immune response, possibly favouring the epithelialization and maintenance of such fistulous tracts (14). Similarly, immunosuppression related to cancer therapy may delay resolution of low-grade infections, allowing the persistence of non-healing cutaneous lesions.

Although the fistula was not directly visible on CBCT due to its soft tissue composition, the imaging was nonetheless valuable in excluding bony or odontogenic involvement. This confirmed the superficial trajectory of the tract and informed our decision to proceed with a minimally invasive surgical approach. Incorporating imaging, even in cases where visualization may be limited, remains essential to exclude deeper or more serious pathology. Alternative diagnostic and management strategies—such as contrast-enhanced imaging, fistulography, or preliminary biopsy—were also considered during the clinical evaluation. However, these were deemed unnecessary given the chronic and superficial nature of the lesion, the absence of any signs of deep tissue or vascular involvement, and the presence of clearly defined intraoral and cutaneous openings. A direct excision under local anesthesia was selected as the safest and most appropriate therapeutic course, especially considering the patient's advanced age and multiple comorbidities. This decision was further validated by the favourable outcome and absence of recurrence at the 6-month follow-up.

Surgical excision remains the definitive treatment for chronic or atypical OCFs. In our case, a complete *en bloc* resection of the tract was performed under local anesthesia with successful outcome and no recurrence at follow-up. This is consistent with the management approach described by Cansız et al., who also reported complete resolution following resection and local flap reconstruction (9).

From a developmental perspective, congenital dermal sinuses and branchial cleft anomalies should be considered in the differential diagnosis, especially when the tract appears epithelialized and contains adnexal structures such as hair follicles (1). Prabhu and Al Abdulla (8) reported a case of recurrent hair growth from Wharton's duct, likely due to heterotopic tissue or incomplete follicle removal, highlighting the persistence of such lesions even after intervention. Although rare, such entities can persist unnoticed into adulthood and become secondarily infected, mimicking odontogenic infections.

Compared to previously published cases, the current report presents several distinct clinical features. For instance, in the case reported by Baughman et al. (1), the presence of intraoral hair was associated with a history of maxillofacial trauma, while Agha-Hosseini et al. (6) described a case of oral hair in a pediatric patient with a history of surgical intervention. In contrast, our patient had no prior trauma, surgery, or dental intervention in the affected area, suggesting a spontaneous origin. Similarly, Nair and Beena (7) reported oral hair associated with a mucosal inclusion cyst, a condition not observed in our case. Furthermore, the location of the fistulous tract in our case emerging from the genian region near fourth quadrant differs from other reports which described midline or submental involvement (1, 6). Most importantly, our case involves oral mucosal inclusion of hair follicles associated with a chronic genian fistula in an elderly polymorbid patient, which, to our knowledge, has not been previously reported in the literature. These comparisons underscore the uniqueness of the current case and its relevance to the differential diagnosis of persistent orocutaneous fistulas.

## 4 Conclusions

This case highlights a rare and diagnostically challenging presentation of a chronic fistula associated with intraoral emergence of a hair follicle. Unlike typical odontogenic fistulas, which often originate from mandibular sources and follow predictable inflammatory pathways, this case involved a non-odontogenic maxillary origin, an epithelialized tract with ectodermal inclusion, and no dental or sinus pathology, exceptionally rare features in the existing literature.

The diagnostic process was further complicated by the patient's polymorbid status, including diabetes, hypothyroidism, ischemic heart disease, and a history of squamous cell carcinoma treated with radio-chemotherapy and immunotherapy. These comorbidities may have contributed to the lesion's persistence, delayed referral, and atypical clinical course.

Successful management was achieved through minimally invasive surgical excision, which remains the treatment of choice in chronic or non-resolving cases, especially when congenital or developmental anomalies are suspected. This case underscores the im-portance of maintaining a broad differential diagnosis when evaluating persistent facial cutaneous lesions and supports a multidisciplinary approach to ensure accurate diagnosis and effective treatment, even in patients with significant systemic comorbidities.

## Data Availability

The original contributions presented in the study are included in the article/Supplementary Material, further inquiries can be directed to the corresponding author/s.
