# Correction: A rare case of chronic genian fistula with oral mucosal hair follicle inclusion: case report and review of the literature

**DOI:** 10.3389/froh.2025.1768742

**Published:** 2026-01-06

**Authors:** Edmond D. Ciora, Mariana I. Miron, Andreea Igna, Andreea G. Miron, Ciprian I. Roi, Mircea Riviș

**Affiliations:** 1 Faculty of Dentistry, University Clinic of Oral Rehabilitation and Dental Emergencies, “Victor Babes” University of Medicine and Pharmacy, Timisoara, Romania; 2 Interdisciplinary Research Center for Dental Medical Research, Lasers and Innovative Technologies, “Victor Babes” University of Medicine and Pharmacy, Timisoara, Romania; 3 Faculty of Dental Medicine, Pediatric Dentistry Research Center, University Clinic of Pediatric Dentistry, “Victor Babes” University of Medicine and Pharmacy, Timisoara, Romania; 4 Faculty of Medicine, “Victor Babes” University of Medicine and Pharmacy, Timisoara, Romania; 5 Pathological Anatomy Resident, Timisoara Municipal Emergency Clinical Hospital, Timisoara, Romania; 6 Research Center of Dento-Alveolar Surgery, Anesthesia and Sedation in Dental Medicine, University Clinic of Anesthesiology and Oral Surgery, “Victor Babes” University of Medicine and Pharmacy, Timisoara, Romania

**Keywords:** case report, rare case, oral hair growth, genian fistula, orocutaneous, non-odontogenic

The funder Victor Babeş University of Medicine and Pharmacy, Timisoara was erroneously omitted.

The original version of this article has been updated.

